# RNA-Seq Analysis of Influenza A Virus-Induced Transcriptional Changes in Mice Lung and Its Possible Implications for the Virus Pathogenicity in Mice

**DOI:** 10.3390/v13102031

**Published:** 2021-10-08

**Authors:** Tianxin Ma, Abdou Nagy, Guanlong Xu, Lingxiang Xin, Danqi Bao, Chenyang Lu, Shiqi Niu, Zihua Wu, Chaochao Ren, Ting Zhang, Jianmei Yang, Qiaoyang Teng, Xuesong Li, Zejun Li, Qinfang Liu

**Affiliations:** 1 Shanghai Veterinary Research Institute, Chinese Academy of Agricultural Sciences, Shanghai 200241, China; matianxin12@163.com (T.M.); baodanqi1@163.com (D.B.); LBJLCY1992@163.com (C.L.); 15837283120@163.com (S.N.); wuzihua6@163.com (Z.W.); sxllengze@126.com (C.R.); tina_zz@outlook.com (T.Z.); yangjianmei@shvri.ac.cn (J.Y.); Tengqy@shvri.ac.cn (Q.T.); LiXuesong@shvri.ac.cn (X.L.); 2 Department of Virology, Faculty of Veterinary Medicine, Zagazig University, Zagazig 44511, Egypt; abdonagy58@rocketmail.com; 3 China Institute of Veterinary Drug Control, Beijing 100081, China; xuguanlongw@163.com (G.X.); xinxeya@sina.com (L.X.)

**Keywords:** influenza A virus, prognosis, macrophages, immune regulation, cell cycle regulation

## Abstract

The influenza A virus (IAV) is an important cause of respiratory disease worldwide. It is well known that alveolar epithelial cells are the target cells for the IAV, but there is relatively limited knowledge regarding the role of macrophages during IAV infection. Here, we aimed to analyze transcriptome differences in mouse lungs and macrophage (RAW264.7) cell lines infected with either A/California/04/2009 H1N1 (CA09) or A/chicken/SD/56/2015 H9N2 (SD56) using deep sequencing. The uniquely differentially expressed genes (UDEGs) were analyzed with the Gene Ontology (GO) and Kyoto Encyclopedia of Genes and Genomes (KEGG) databases; the results showed that the lungs infected with the two different viruses had different enrichments of pathways and terms. Interestingly, CA09 virus infection in mice was mostly involved with genes related to the extracellular matrix (ECM), while the most significant differences after SD56 infection in mice were in immune-related genes. Gene set enrichment analysis (GSEA) of RAW264.7 cells revealed that regulation of the cell cycle was of great significance after CA09 infection, whereas the regulation of the immune response was most enriched after SD56 infection, which was consistent with analysis results in the lung. Similar results were obtained from weighted gene co-expression network analysis (WGCNA), where cell cycle regulation was extensively activated in RAW264.7 macrophages infected with the CA09 virus. Disorder of the cell cycle is likely to affect their normal immune regulation, which may be an important factor leading to their different prognoses. These results provide insight into the mechanism of the CA09 virus that caused a pandemic and explain the different reactivities of monocytes/macrophages infected by H9N2 and H1N1 IAV subtypes.

## 1. Introduction

Influenza is a seasonal disease that causes yearly winter epidemics globally. Influenza viruses are enveloped negative-stranded RNA viruses with segmented genomes containing eight gene segments. The influenza virus belongs to the Orthomyxoviridae family and consists of four distinct genera: influenza A, B, C, and D viruses. IAVs have a wide range of hosts, including humans, birds, pigs, horses, and marine mammals [1]. IAVs can be divided into 18 HA subtypes and 11 NA subtypes according to their HA and NA proteins. The pathogenicity of the IAV varies between distinct subtypes [2]. Four major influenza pandemics have occurred in the past 100 years: the H1N1 Spanish influenza in 1918, the H2N2 Asian influenza in 1957, the H3N2 Hong Kong influenza in 1968, and the H1N1 swine influenza in 2009 [3].

In April 2009, an H1N1 influenza virus (A(H1N1) pdm09) emerged in North America. This virus quickly spread to more than 30 countries, leading to the first influenza pandemic in the 21st century [4,5]. Previous studies showed that A(H1N1) pdm09 induced higher levels of proinflammatory cytokines, making it more virulent [6]. Meanwhile, A(H1N1) pdm09 induced lower levels of innate immune response and antiviral and pro-inflammatory cytokine mRNA than seasonal influenza strains in macrophages and dendritic cells [7,8]. The pathogenesis of A(H1N1)pdm09 still remains largely unclear.

H9N2 is an avian influenza virus with a great impact on public health. It is the most prevalent subtype of avian influenza A virus in birds in China, which poses a significant threat to the poultry industry and public health [9,10,11]. Although the H9N2 subtype has low pathogenicity, it has a wide spectrum of hosts. Studies showed that most of the currently circulating H9N2 strains prefer to bind to SA-ɑ-2,6-Gal receptors, which are mainly expressed in human airway epithelial cells [12,13,14]. Surveillance data indicated that about 2.3–4.6% of poultry workers had antibodies against H9N2, and 20.21–44.85% of dogs were seropositive to H9N2 in Southern China [15,16,17]. Although the H9N2 influenza virus replicates efficiently in mouse models, it is not lethal and has a relatively low virulence in mice, the underlying mechanism of which remains unknown.

In this study, an H9N2 IAV subtype A/chicken/SD/56/2015 H9N2 (SD56) strain replicated efficiently in mouse lungs without causing any clinical signs. In contrast, the A(H1N1) pdm09 A/California/04 2009 (CA09) strain replicated efficiently in mouse lungs and caused 100% mortality in mice. However, the underlying mechanism of the different virulence between these two viruses remains unknown. RNA-Seq is a powerful approach that can provide precise measurements of the levels of transcripts and their isoforms [18]. In this study, conjoint RNA-Seq analysis was performed on the lungs and macrophages infected with CA09 and SD56, respectively. These results contribute toward understanding the mechanisms of the different virulences of various subtype influenza viruses.

## 2. Materials and Methods

### 2.1. Cell Lines and Viral Propagation

MDCK and RAW264.7 cells were purchased from the American Type Culture Collection (ATCC; #TIB-71 and #CCL-34). These cells were maintained in Dulbecco’s modified Eagle’s medium (DMEM) supplemented with 10% fetal bovine serum (FBS; PAN Biotech, Aidenbach, Germany) at 37 °C with 5% CO_2_. Cells were cultured until the confluency reached approximately 80%. The A/California/04/2009 H1N1 (CA09) and A/chicken/SD/56/2015 H9N2 (SD56) were preserved at the Shanghai Veterinary Research Institute of the Chinese Academy of Agricultural Sciences (SHVRI, CAAS). These viruses were propagated in 9- to 11-day-old specific-pathogen-free (SPF) egg embryos. The 50% tissue culture infective dose (TCID_50_) of the harvested viruses was determined using MDCK cells.

### 2.2. Viral Infection

For the in vitro infections, RAW264.7 cells were seeded into 100 mm cell culture dishes and were cultured overnight until the cells reached approximately 80% confluency. The RAW264.7 cells were infected with either CA09 or SD56 (MOI = 10). Infected cells were incubated at 37 °C with 5% CO_2_ for 2 h, then the medium was replaced with fresh serum-free medium containing proper concentrations of tosylsulfonyl phenylalanyl chloromethyl ketone (TPCK)-treated trypsin. In addition, the uninfected RAW264.7 cells served as a negative control. Total RNA was extracted from the infected and control RAW264.7 cells collected at 12 h post infection for RNA-Seq and transcriptional analysis. All experiments were repeated with biological triplicates.

For the in vivo infections, five-week-old female BALB/c mice were divided into three groups (*n* = 12 mice per group). Mice of two infection groups were inoculated with either CA09 or SD56 intranasally (10^6^ TCID_50_/mouse). The mice in the control group were inoculated with PBS. Three mice from each group were necropsied and lungs were collected after 3 and 5 days post infection (dpi). Lungs were collected, homogenized, and subjected to virus titration and RNA extraction. The remaining mice were observed for clinical signs, and their body weight was measured until 14 dpi. Total RNA was extracted from 3 mouse lungs collected after 3 dpi for RNA-Seq and transcriptional analysis. All animal studies in this research were conducted in accordance with the guidelines of the Animal Care and Use Committee of Shanghai Veterinary Research Institute, and all animal study protocols were approved by the Chinese Academy of Agricultural Science (permit number: SHVRI-Po-0120).

### 2.3. Library Construction and RNA Sequencing

Total RNA was extracted from the infected and negative control samples using TRIzol (Invitrogen, San Diego, CA, USA) according to the manufacturer’s instructions. The concentration and purity of the RNA were detected using a Nanodrop2000 (Thermo Fisher Scientific, NYC, New York, NY, USA). RNA integrity was detected using 1% agarose gel electrophoresis, and the RIN value was determined using an Agilent 2100 (Agilent Technologies, Santa Clara, CA, USA). Total RNA was utilized to isolate the mRNA via A–T base pairing with magnetic beads with Oligo(dT). After the fragmentation buffer was added, mRNA was broken randomly and small fragments of about 300 bp were separated through magnetic bead screening. Sequencing libraries were prepared using a Truseq™RNA Sample Prep Kit (Illumina, San Diego, CA, USA) according to the manufacturer’s recommendations. After the fragment screening and library enrichment, the final library was obtained.

The RNA-Seq protocol was provided by Shanghai Majorbio Bio-Pharm Technology Co., Ltd., Shanghai, China. First, the sequences of the constructed library were quantified using a Quantus™ Fluorometer and a QuantiFluor^®^ dsDNA System (Promega, Madison, WI, USA). Then, after mixing the data in the appropriate proportion in the machine, bridge PCR amplification was performed on cBot to generate clusters. Library extraction was achieved by Agencourt AMPure XP (Beckman Coulter, CA, USA). Finally, sequencing was performed on an Illumina Hiseq NovaSeq6000 sequencer (Illumina, San Diego, CA, USA) using HiSeq X Reagent Kits and NovaSeq Reagent Kits (Illumina, San Diego, CA, USA).

### 2.4. Transcriptome Assembly and Reads Mapping

Adaptor sequences, joint sequences, and long/short sequences that affect the quality of subsequent assembly were filtered out from the raw data using the software SeqPrep (https://github.com/jstjohn/SeqPrep (accessed on 21 April 2021)) and Sickle (https://github.com/najoshi/sickle (accessed on 21 April 2021)). Briefly, joint sequences, bases with low mass (mass value less than 30) at the end of the sequence, reads containing poly-N > 10%, adapters and the quality-pruned sequences that were less than 50 bp in length were removed. The trimmed clean data were mapped to the *Mus musculus* genome (Ensembl, http://www.ensembl.org/Mus_musculus/Info/Index (accessed on 21 April 2021)). Based on the selected reference genome sequence, the mapped reads were spliced and gene expression levels were calculated using StringTie (http://ccb.jhu.edu/software/stringtie/ (accessed on 21 April 2021)) or Cufflinks (http://cole-trapnell-lab.github.io/cufflinks/ (accessed on 21 April 2021)).

### 2.5. Expression and Function Annotation Analysis

The expression level of each transcript was calculated according to the fragments per kilobase of exon per million mapped reads (FRKM) method. RSEM (http://deweylab.github.io/RSEM/ (accessed on 21 April 2021)) was used to quantitatively analyze the expression levels of genes for subsequent analysis of the DEGs among different samples with the quantitative index transcript per million (TPM) [19,20].

The genes were annotated and classified based on six databases (NR, swiss-prot, Pfam, EggNOG, GO, and KEGG). DIAMOND software was used to compare the sequences of the genes with the NR, swiss-prot, and EggNOG databases. BLAST2GO was used to annotate and analyze sequences with the GO database. HMMER software was used to make alignments with the Pfam database. KEGG Orthology results were obtained using KOBAS2.1. In the above analysis, the e-value was less than 0.00005.

### 2.6. GO Enrichment and KEGG Pathway Analysis

GO is a database that was established by the genetic ontology association in which genes can be classified mainly in terms of three aspects: biological process, cellular component, and molecular function. The software Goatools was used to analyze the GO enrichment and function of the DEGs. By annotating the KEGG database, the corresponding gene KO number and the related biological pathways involved with these genes were identified. Fisher’s accurate test was used in the GO enrichment and KEGG pathway analysis. When the adjusted *p*-value (*p*-adjust) < 0.05, the GO function/KEGG pathway was considered to be significantly enriched [21].

### 2.7. Gene Set Enrichment Analysis (GSEA)

GSEA was conducted with GSEA version 3.0, which was downloaded from the Broad Institute (http://www.broadinstitute.org/gsea/downloads.jsp) (accessed on 21 April 2021)[22]. The target gene set was constructed according to the gene function annotation and arranged according to the expression differences between the two types of samples. Then, whether the gene set was enriched at the top or the bottom of the arranged table was tested. By detecting the expression differences of the a priori gene set, this analysis can detect genes with insignificant differences but significant biological differences at the overall level without specifying a cutoff threshold for differential expression. For the analysis results, enriched gene sets were assigned based on |NES| > 1, NOM *p*-value < 0.05, and FDR q-value < 0.25.

### 2.8. Construction of a Scale-Free Co-Expression Network Using WGCNA

WGCNA (https://horvath.genetics.ucla.edu/html/CoexpressionNetwork/Rpackages/WGCNA/) (accessed on 21 April 2021) is a common approach that is used to identify highly correlated gene clusters/modules that contain genes with common expression patterns from different samples, where the co-expression relationship between genes is generally measured using the expression correlation coefficient between them [23,24]. The set of genes that were classified as having similar expression patterns is called a cluster/module. After obtaining generic expression modules, the modules are then related to biological traits of concern (various infection types) to explore the association between gene networks and phenotypes, as well as the key hub genes in the network.

### 2.9. Validation of RNA-Seq Data

Total RNAs of cell samples and lung tissues that were infected with the influenza virus, as well as control samples, were extracted according to the same protocol. A subset of 11 genes with annotations from a statistical analysis of the RNA-Seq was selected for quantitative real-time RT-PCR (qRT-PCR) analysis, where the primers are shown in Table 1. Briefly, cDNAs were synthesized using Oligo(dT) and M-MLV Reverse Transcriptase (Promega, Madison, WI, USA) according to the manufacturer’s instructions. qRT-PCR was performed using the SYBR PrimeScript kit (TaKaRa Bio Inc., Tokyo, Japan) and Applied Biosystems™ QuantStudio™ 5 Real-Time PCR System. Primer sequences that were used for the RT-qPCR are presented in Table 1. The gene expression values were calculated according to the 2^−ΔΔCT^ method [25], and the results are presented as the log_2_ fold change.

## 3. Results

### 3.1. Pathogenicity of CA09 and SD56 in Mice

**Figure 1:**
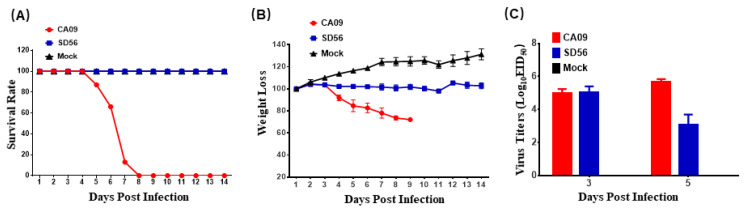
Pathogenicity of CA09 and SD56 in mice. Mice (*n* = 12 per group) inoculated with CA09, SD56, or PBS were monitored for mortality and the change in body weights for 14 days (once per day) after infections. (**A**) The survival rates of the infected mice. (**B**) The changes in body weights of the infected mice. (**C**) The lungs of each group were harvested at 3 and 5 dpi and the lung viral loads were titrated on SPF embryos.

To compare the pathogenicity of CA09 and SD56, five-week-old BALB/c mice were infected with CA09 and SD56, respectively. The CA09 infection caused 100% mortality in the infected mice, but the mice in the SD56-infected group did not show any clinical signs until the end of the study (Figure 1A). The mouse body weight results showed that CA09 caused significant weight loss compared with the SD56 and mock groups (Figure 1B). The virus load data indicated that both CA09 and SD56 replicated to a similar level in mice lungs after 3 dpi, while the CA09 replicated to higher titers than SD56 after 5 dpi (Figure 1C), which indicated that CA09 was more virulent than SD56 in the mouse model.

### 3.2. Global Transcriptome Changes Induced by IAV Infection in Lungs and RAW264.7 Cells

A total of 18 cDNA libraries were constructed and sequenced on Illumina Hiseq and NovaSeq6000, where 906 million raw reads were generated. A total of 894 million (98.68% of the raw reads) clean reads were obtained. On average, 49.71 million clean reads were obtained for each sample. Among the 18 samples, 88.78–96.10% of the clean reads were mapped to the reference genome (*Mus musculus*) and the reads distributions in chromosomes were similar between the samples (Figure S1A). Moreover, 83.07–90.94% of the clean reads were uniquely mapped corresponding to the CDS region (Table S1). There was no obvious bias in the coverage of the sequencing data, which showed that the sequences that were obtained were evenly distributed on the *Mus musculus* genome (Figure S1B).

**Figure 2:**
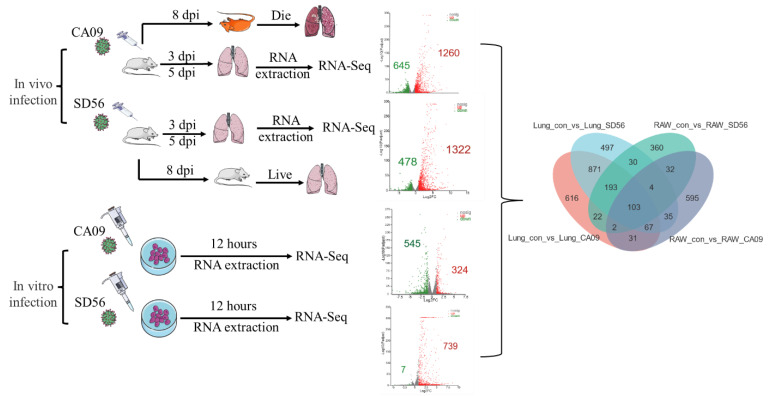
Sample processing procedures and global transcriptome changes. In the volcano plot, the splashes were for different genes, among which, grey dots represent genes with no significant discrepancy, red dots represent genes that were significantly upregulated, and green dots represent significantly downregulated genes. In the Venn diagram, the numbers indicate unique and common DEGs in four groups.

To analyze DEGs after infection, online software DESeq2 and RSEM were utilized for the gene differential expression analysis with FC ≥ 2/FC ≤ 0.5 (log_2_ fold-change ≥ 1/log_2_ fold-change ≤ −1) and a *p*-adjust value < 0.05. As depicted in Figure 2, the volcano plot illustrates that, compared with the control lung samples, 1260 genes were upregulated and 645 genes were downregulated in CA09-infected lungs; 1322 genes were upregulated and 478 genes were downregulated in SD56-infected lungs. Following the same parameters, there were 324 significantly upregulated genes and 545 significantly downregulated genes in CA09-infected RAW264.7 samples. As for the RAW264.7 cells infected by SD56, there were 739 significantly upregulated genes and only 7 significantly downregulated genes (Figure 2).

### 3.3. SD56 Infection Stimulated a Stronger Immune Response Than CA09 in Mice Lungs

**Figure 3:**
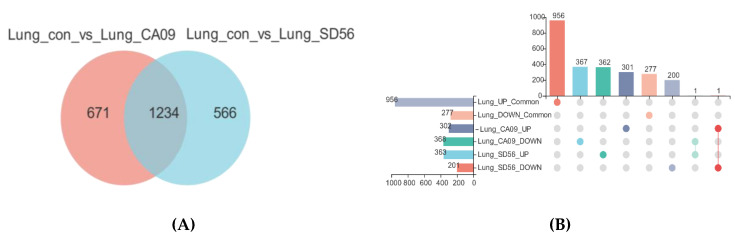
Summary of differentially expressed genes (DEGs) among the CA09 and SD56 infected samples. (**A**) Venn diagram of common lung DEGs when comparing the two groups (con vs. CA09 and con vs. SD56). (**B**) Upset diagram of uniquely DEGs upon CA09 and SD56 infection.

To explore the mechanism of the pathogenicity difference between SD56 and CA09, a Venn analysis of the infected mouse lung tissues was performed. Among all the DEGs in the lung samples, 1234 DEGs were common in two infected groups, 671 genes were only differentially expressed in CA09-infected lungs, and 566 genes were uniquely differentially expressed in SD56-infected lungs (Figure 3A). The Upset diagram in Figure 3B shows the specific change trends of two groups of DEGs: among 671 uniquely differentially expressed genes in CA09-infected lungs, 301 genes were upregulated and 367 genes were downregulated; among 566 uniquely differentially expressed genes in SD56-infected lungs, 362 genes were upregulated and 200 genes were downregulated (Figure 3B).

**Figure 4:**
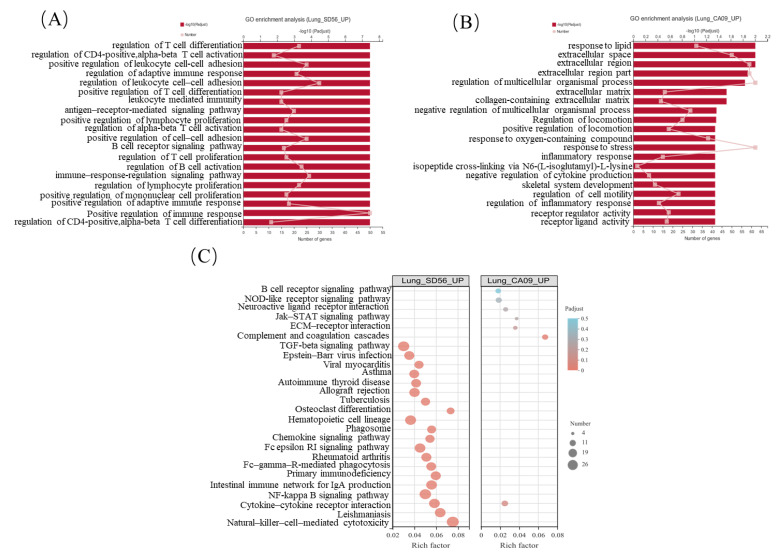
GO and KEGG functional enrichment of upregulated genes in mice lungs. (**A**) GO enrichment analysis of uniquely upregulated lung genes in SD56 relative to the control; (**B**) GO enrichment analysis of uniquely upregulated lung genes in CA09 relative to the control; (**C**) KEGG enrichment bubble diagram of uniquely upregulated lung genes in SD56 relative to the control (left) and in CA09 relative to the control (right). Circles indicate the numbers of genes and colors depict the richness factors.

GO and KEGG enrichment analyses were further performed to identify the putative functions of uniquely DEGs in these unique gene sets. In the set of SD56 uniquely up-regulated genes, the regulation of T cell differentiation, regulation of adaptive immune response, regulation of B cell activation, and other terms related to immune activation were significantly enriched. Almost all the top 20 enriched terms referred to the immune system (Figure 4A). On the other hand, in CA09 uniquely upregulated genes, the enriched terms had no significant correlation with immune regulation (Figure 4B). A similar result was observed from the KEGG enrichment bubble diagram in Figure 4C, where uniquely upregulated pathways in lungs after an SD56 infection mainly involved natural-killer-cell-mediated cytotoxicity (map04650), the NF-kappa B signaling pathway (map04064), and the B cell receptor signaling pathway (map04662); uniquely upregulated pathways of CA09-infected lungs had almost no correlation with immune regulation. 

According to the GO and KEGG enrichment analyses of the uniquely upregulated genes of CA09 and SD56 infections, SD56 infections were found to have specifically activated the adaptive immune response and a large number of immune-related terms were upregulated, while CA09 infections activated different pathways compared with SD56, where the genes enriched in the CA09 infection were concentrated in other pathways.

### 3.4. CA09 Infection Significantly Regulated ECM-Related Pathways in Mouse Lungs

**Figure 5:**
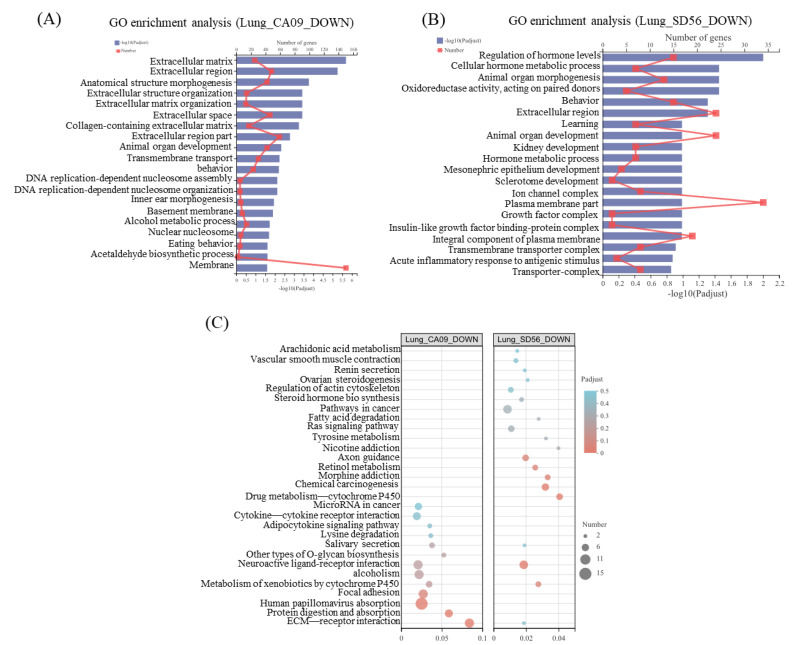
GO and KEGG functional enrichment of downregulated genes in mice lungs. (**A**) GO enrichment analysis of uniquely downregulated lung genes in CA09 relative to the control; (**B**) GO enrichment analysis of uniquely downregulated lung genes in SD56 relative to the control; (**C**) KEGG enrichment bubble diagram of uniquely downregulated lung genes in SD56 relative to the control (left) and in CA09 relative to the control (right). Circles indicate numbers of genes and colors depict the richness factor.

Since the CA09 infections did not cause significant upregulation of the immune regulation, to explore the mechanism of lethality in mice infected with CA09, the uniquely upregulated and downregulated genes in CA09-infected mice lungs were analyzed. Based on a GO enrichment analysis, CA09 uniquely upregulated genes were most significantly enriched in the terms of lipid, extracellular space, and extracellular region (Figure 4B). In addition, CA09 uniquely downregulated genes were related to pathways of the extracellular matrix, extracellular region, extracellular space, and other extracellular related terms (Figure 5A). 

Similarly, in the KEGG analysis of CA09-infected lungs, both uniquely upregulated and downregulated genes were enriched in terms of the ECM–receptor interaction (map04512) and pathways associated with this signaling pathway (Figure 5C). Further analysis showed that the ECM-related genes that were upregulated after CA09 infections mainly included metalloenzymes, such as Mmp9 (a member of the matrix metalloproteinases), Adamts15 (proteases containing a prometallo protease domain), Calca (functions as a vasodilator), Ctsk (a member of the peptidase C1 protein family), and other genes that impair the cellular junction and morphology of cells. Meanwhile, ECM-related genes that maintain intercellular connectivity and morphology were mainly downregulated after CA09 infections, such as CoL10A1 (collagen), Pcdh12 (adhesion molecules), and Gdf5 (growth differentiation factor). Furthermore, the genes that were uniquely downregulated in SD56 infections mainly included terms that are associated with the regulation of hormone levels, cellular hormone metabolic process, animal organ morphogenesis, and oxidoreductase activity (Figure 5B). All the results indicated that the CA09 infections impaired the cellular junction in lung tissue, increased vascular permeability, and loss of a normal cytoskeleton, which might lead to the aggravation of infection and poor prognosis of the infected mice.

### 3.5. Abnormal Regulation of Macrophage Cell-Cycle-Related Genes after CA09 Infection

Macrophages, as a kind of important immune cells, are involved in congenital immune responses and can activate the adaptive immune response. To explore transcription profile changes of macrophages infected with IAV, DEGs in influenza-virus-infected RAW264.7 cells were analyzed (both CA09 and SD56 caused obvious cytopathic effects in RAW264.7 cells, as shown in Figure S2). GO enrichment analysis showed that terms related to cell migration, cell adhesion, and cell differentiation were mainly enriched in CA09-infected macrophages, and terms related to immune regulation were mainly enriched after SD56 infections (Figure S3).

**Figure 6:**
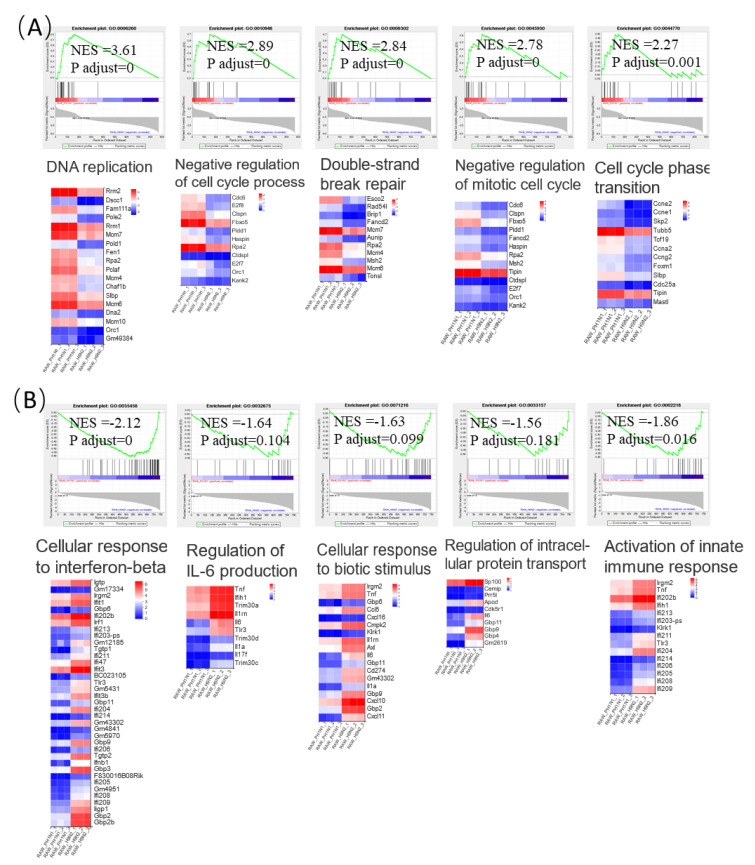
GSEA and expression heatmap. Ten representative significantly enriched gene sets from the GSEA of the RAW264.7 RNA-Seq data. (**A**) Enrichment plots comparing CA09 with PBS were depicted with five sets of genes; (**B**) enrichment plots comparing SD56 with PBS were depicted with five sets of genes, demonstrating the expression heatmap signatures of genes involved in the GSEA. Comparison of samples, NES, nominal *p*-value, and FDR q-value were determined using the GSEA software and are indicated within each enrichment plot.

Due to the limitation of GO enrichment on detecting global changes of gene sets rather than individual genes, GSEA was carried out to study a functional analysis of the interaction within all DEGs. Among the 869 DEGs obtained from the RAW264.7 cells that were infected with CA09, we unexpectedly found that all enriched genes were relevant to the regulation of cell cycle, including DNA replication, negative regulation of cell cycle process, double-strand break repair, negative regulation of mitotic cell cycle, and cell cycle phase transition (Figure 6A). In addition, it was noticed that the enriched terms of 746 DEGs in SD56-infected cells were remarkably different than those of the CA09-infected cells, which mainly included immune defense and antiviral association terms that were enriched, such as cellular response to interferon-beta, activation of the innate immune response, regulation of interleukin-6 production, cellular response to biotic stimulus, and regulation of intracellular protein transport (Figure 6B).

All the results of the GSEA indicated that SD56 infections caused significantly activated immune responses of macrophages, but not in CA09-infected macrophages, where cell cycle regulation was extensively activated. The inability to perform immune-modulatory functions might have led to the abnormal regulation of the cell cycle in macrophages.

### 3.6. Weighted Gene Co-Expression Network Analysis Revealed the Hub Genes in CA09- and SD56-Infected Macrophages

**Figure 7:**
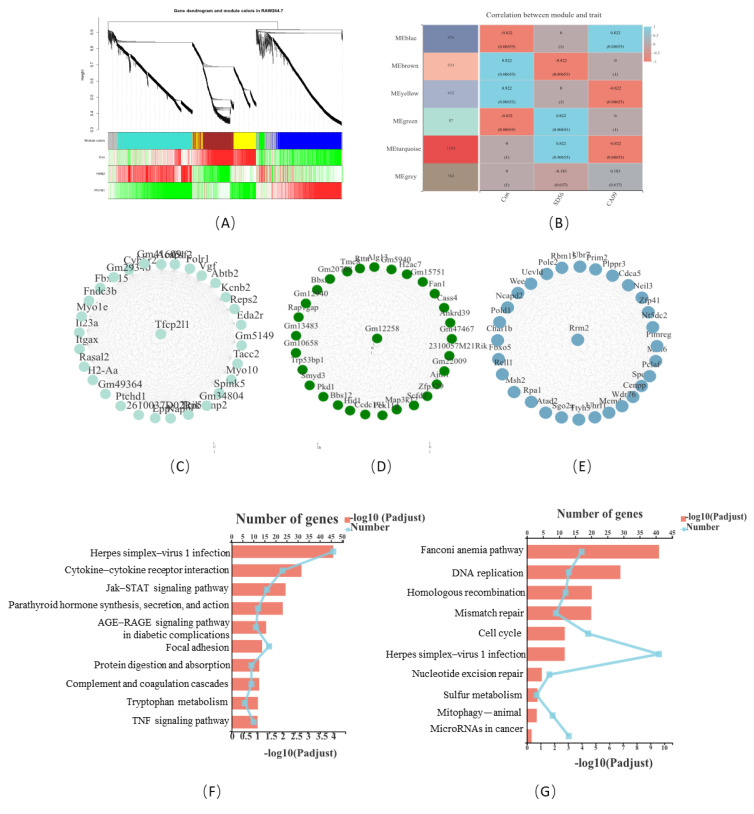
WGCNA of the expression data. (**A**) Gene co-expression modules. Genes that could not be clustered into one of these modules were assigned to the grey module. Different colors represent different modules. Each row represents a phenotype, and each column represents a gene in the module. (**B**) Module–trait relationship. The top value in each square shows the correlation between the module eigengene and the outcome. The bottom value is the *p*-value of each correlation. (**C**–**E**) Visualization of the three modules that were highly correlated with infections with a *p*-value less than 0.05 using Cytoscape. (**F**,**G**) KEGG enrichment of the different modules.

To further verify the pivotal functional DEGs of the macrophages that were infected with two subtypes of the IAV, WGCNA was used to classify the DEGs. The genes were clustered into six modules (ME) (Figure 7A). Figure 7A shows the correlations between the genes and phenotypes (CA09 infection, SD56 infection, and mock) in each module. It was noticeable that the SD56 infections and CA09 infections corresponded to different modules. The DEGs in the SD56 infections were mainly located in ME green and ME turquoise (ME correlation= 0.822, *p*-value = 0.00655), and the DEGs in the CA09 infections mostly corresponded to ME blue (ME correlation= 0.822, *p*-value = 0.00655) (Figure 7B). The hub gene of the module was obtained through visual network analysis, the correlation within the module was calculated using the Spearman coefficient, and a network diagram of each module was obtained by Cytoscape software. The hub genes in different modules are shown in Figure 7C–E.

We focused on the function of modules that correspond to two virus subtypes in the infected groups. Since the MEgreen and MEturquoise had a close correlation with SD56 infection, we combined the genes of those two modules (named MEGREEN module) and performed KEGG pathway enrichment analysis. According to the KEGG enrichment assay, genes in MEgreen and MEturquoise enriched in the KEGG pathways were associated with Herpes simplex virus 1 infection (involved in MHC-I- and MHC-II-mediated antigen presentation, as well as BCL2-mediated anti-apoptotic effect (Figure S4A)), cytokine–cytokine receptor interaction, Jak–STAT signaling pathway, and others (Figure 7F). Taking the same approach, genes in the MEblue module were most associated with the pathway of Fanconi anemia (mainly regulates the mismatch repair and homologous recombination (Figure S4B)), DNA replication, and the cell cycle, as shown in Figure 7G.

According to the WGCNA, the major factors of the CA09 infection of macrophages were the changes in the cell cycle and DNA damage repair, while the most critical pathways of SD56 infection of macrophages were the MHC-mediated antigen presentation and anti-apoptosis.

### 3.7. Quantitative Real-Time PCR Validation of RNA-Seq Data 

**Figure 8:**
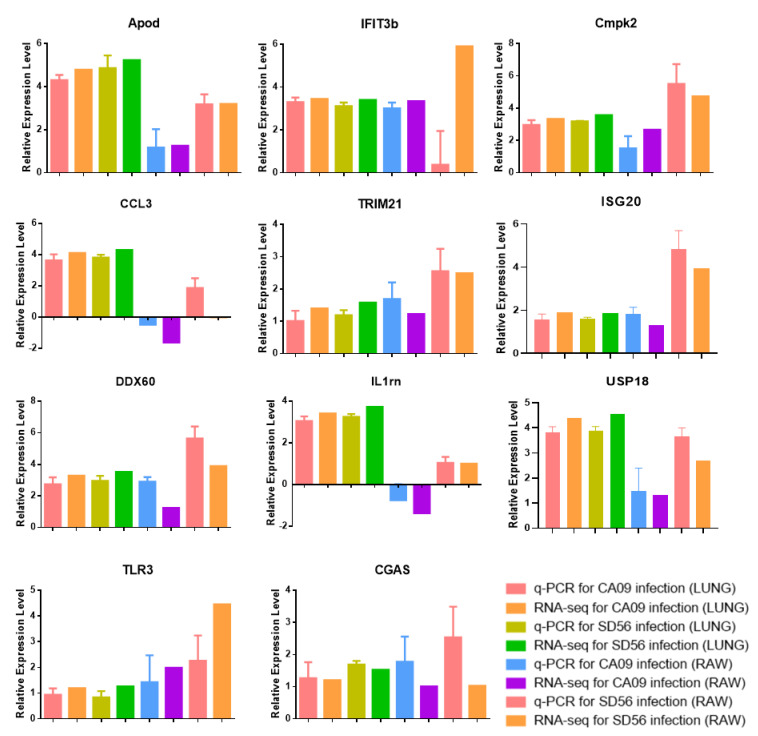
Verification of the relative expression levels using qPCR. Expression levels of selected differentially expressed genes that were associated with IAV infection in the lungs and RAW264.7 cells were measured using qPCR. The expression levels were normalized to the actin expression.

To verify the results of transcriptome sequencing, a total of eleven genes were selected for the qRT-PCR. As shown in Figure 8, the expression levels of APOD, IFIT3B, CMPK2, TRIM21, ISG20, DDX60, USP18, TLR3, and CGAS were upregulated in CA09/SD56 infected samples, CCL3 and IL1RN were downregulated in CA09-infected RAW264.7 cells and upregulated in other samples. The qRT-PCR validation showed that the expression levels of the selected genes were consistent with the RNA-Seq; therefore, the transcriptome sequencing results were reliable

## 4. Discussion

Both CA09 and SD56 replicated efficiently in mouse lungs, where there were no significant differences in the viral titers at 3 dpi. However, the CA09 infection caused 100% mortality in the infected mice, while the mice in the SD56-infected group only showed slight weight loss. The mechanism of the different pathogenicities the two viruses exhibited in mice remains unknown, thus the transcriptomes difference in infected lungs and RAW264.7 cells were evaluated to explore the underlying mechanism. There were a large number of DEGs between the CA09- and SD56-infected groups. The GO analysis showed that the main DEGs in the CA09-infected lungs were related to the lipids, extracellular space, extracellular region, and extracellular matrix. Meanwhile, in the SD56-infected lungs, the upregulated GO terms were almost all related to immune regulation, such as the regulation of T cell differentiation, regulation of the adaptive immune response, regulation of B cell activation, and others. KEGG enrichment analysis of lungs infected with SD56 viruses yielded similar results as the GO enrichment, where pathways associated with natural-killer-cell-mediated cytotoxicity, the NF-kappa B signaling pathway, and the B cell receptor signaling pathway were uniquely enriched in upregulated DEGs of SD56 infected lungs. Activation of the immune-related response in SD56-infected lungs might have contributed to the low pathogenicity of SD56 in mice. The present results suggested that activation of the immune response, the maintained homeostasis, and the inhibition of metabolism played an important role in controlling the disease outcome of SD56-infected mice.

The main DEGs in CA09-infected lungs were the ECM-related genes that maintain intercellular connectivity and cell morphology. Studies showed that during an IAV infection, ECM proteolysis plays a role in protecting tissue integrity in the lungs [26]. The ECM consists of a complex mixture of structural and functional macromolecules and serves an important role in tissue and organ morphogenesis and in the maintenance of the cell and tissue structure and function. Specific interactions between cells and the ECM are mediated by transmembrane molecules, mainly integrins and perhaps also proteoglycans, where these interactions lead to direct or indirect control of cellular activities, such as adhesion, migration, differentiation, proliferation, and apoptosis. Downregulation of the ECM composition and structure is associated with the development and progression of several pathologic conditions [27] and cancer-related diseases [28]. Considering the importance of the ECM for homeostasis maintenance, disruption of the intercellular junctions leads to increased paracellular permeability, which might contribute to the virulence of CA09 in mice.

The lung is a complex tissue, where alveolar macrophages play an important role in the defense against infection due to viruses, bacteria, mycobacteria, fungi, etc. [29,30,31]. However, the responses of macrophages in different subtypes of IAV infection remain largely unclear. Previous studies showed that the highly pathogenic H5N1 virus induced higher levels of cytokines in macrophages, resulting in inflammatory infiltration of the lungs [29]. The transcriptomics analysis in this study showed that CA09 infection did not induce upregulation of inflammatory cytokines in RAW264.7 cells. In contrast, the cell-cycle-associated genes of RAW264.7 cells were negatively regulated by CA09 infection, which might have contributed to the lethality of CA09. Manipulating the cell cycle has been used by many viruses to facilitate virus replication, where studies were reported showing that A/WSN/33 (H1N1) induced G0/G1 cell cycle arrest through inhibiting the expression and activity of RhoA protein by NS1 [32]. Another study reported that the influenza virus protein M1 interacts with SLD5 to block the host cell cycle [33,34]. While GSEA showed that SD56 infection mainly stimulated immune regulation in RAW264.7 cells, which is consistent with the results in the lungs., the genes related to innate immune response, i.e., the cellular response to IFN-β, were enriched and the IL-6 related pathway was upregulated in SD56-infected cells. IL-6 plays a critical role in both the innate and adaptive immune responses that protect the host from pathogens. Studies showed that IL-6 also enhanced virus clearance and the immune cell response during influenza infection [34]. Through the WGCNA, it was found that the macrophages infected with CA09 and SD56 showed two different gene expression patterns. The DEGs in the RAW264.7 cells that were infected with CA09 suggested that genes related to DNA damage repair were hub genes in CA09 infection, and the genes related to antigen presentation and anti-apoptosis were hub genes in SD56 infection. All the results suggested that the DEG patterns that were caused by the CA09 and SD56 might contribute to the distinct pathogenicity of these two viruses in mice.

In conclusion, our study presented the different DEG patterns caused by CA09 and SD56 in vivo and in vitro. CA09 infection mainly induced ECM-related gene regulation in the lungs and cell cycle disorder in macrophages cells, resulting in disruption of the intercellular junctions, increased inflammatory cell infiltration, and disfunction of macrophages, which might have contributed to the high virulence of CA09 in mice. In contrast, H9N2 infection mainly stimulated the immune response, both in vivo and in vitro, which enhanced the antiviral immunity of the infected mice and resulted in early virus clearance.

## Figures and Tables

**Table 1:** Primer sequences that were used for the RT-qPCR.

Gene Name	Forward Primer (5′–3′)	Reverse Primer (5′–3′)
Apod	GCCACCGACTATGAGAACTATG	CACTGTTTCTGGAGGGAGATTAG
IFIT3b	CCTCAGAACCAGTACGTGAAAG	GGAGGACATCCGTTTGATTAG
Cmpk2	CCTGCTCAAACCTGACCTTATC	GGCCTCAAGTTCTGCTTCTT
CCL3	GAAGATTCCACGCCAATTCATC	GATCTGCCGGTTTCTCTTAGTC
IL1rn	TTGTGCCAAGTCTGGAGATG	CTCAGAGCGGATGAAGGTAAAG
TRIM21	GATAGCCCAGAATACCAAGAAGAG	GCCCATCTTCCTCACAGAATAG
DDX60	GGTATCCCGATTGGCTGATATG	GAGACACAAGTGGCGAATCT
TLR3	ACCTCCAGAAGAACCTCATAAC	GAACGGATTGAAGCGCATATC
CGAS	GGAACCGGACAAGCTAAAGA	CAGGCGTTCCACAACTTTATTC
ISG20	GGAGAGATCACGGACTACAGAA	TAGCCTGGCTTCACCAAATG
USP18	AGAGGACCATGAAGAGGAAGA	CGTCTGTCCGATGTTGTGTAA
ACTIN	CCGTAAAGACCTCTATGCCAAC	AGGAGCCAGAGCAGTAATCT

## Supplementary Materials

The following are available online at https://www.mdpi.com/article/10.3390/v13102031/s1, Figure S1: Transcriptome quality assessment, Table S1: Transcriptome profile of 18 cDNA libraries. Figure S2: Cytopathic effect of RAW264.7 cells infected with CA09 and SD56. Figure S3: GO functional enrichment of differentially expressed genes in RAW264.7 cells. Figure S4: Details of the signaling pathways involved in the modules.
