# Supplementary material for: RNA-Seq Analysis of Influenza A Virus-Induced Transcriptional Changes in Mice Lung and Its Possible Implications for the Virus Pathogenicity in Mice
Source: Viruses. 2021 Oct 8;13(10):2031. doi: 10.3390/v13102031 (PMC8538690; doi:10.3390/v13102031)
Supplement: Supplementary file 1 [file viruses-13-02031-s001.zip › viruses-1387224-supplementary.pdf]

viruses

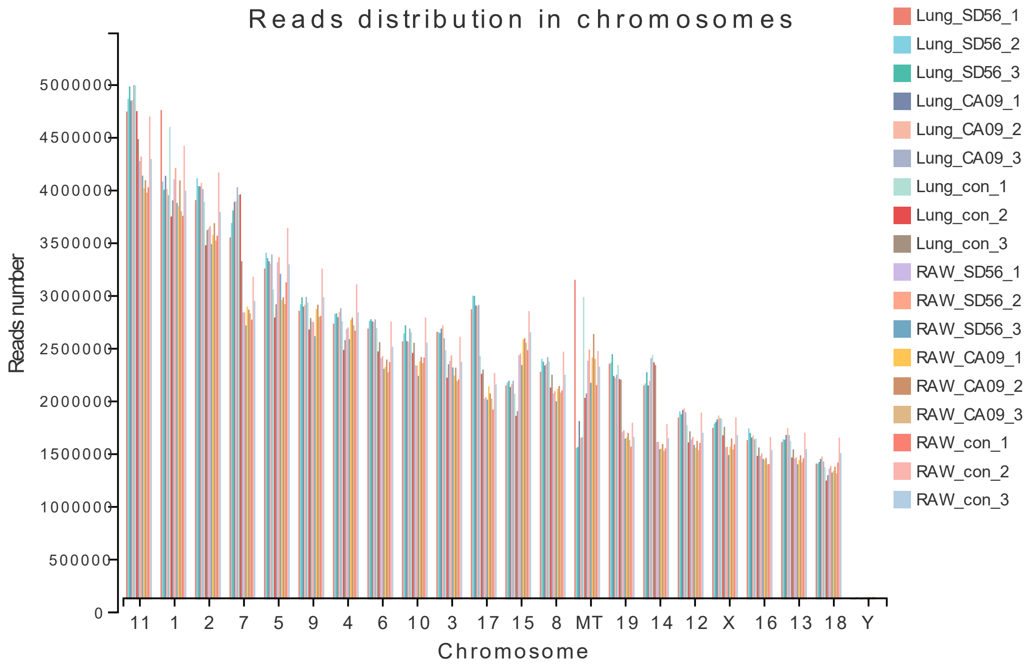

(A)

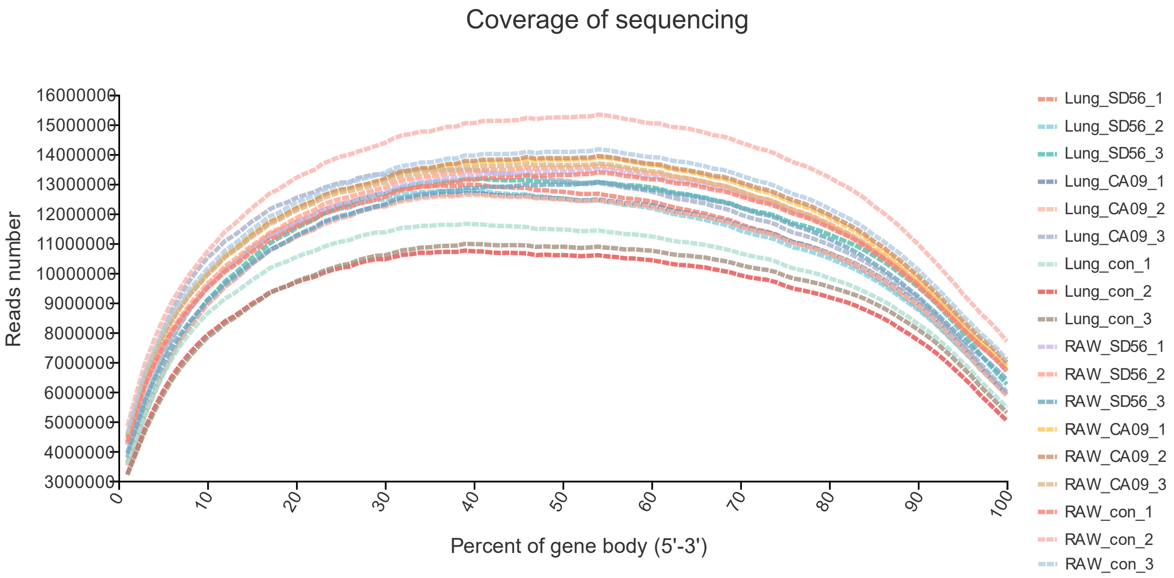

(B)

Figure S1. Transcriptome quality assessment. The quality of the transcriptome sequencing was assessed using mapped reads. (A) The number of sequences on chromosomes was statistically compared to reflect the distribution of the sequences on each chromosome. (B) Sequencing coverage analysis, the curve reflects whether the sequence obtained by sequencing is evenly distributed on the gene.

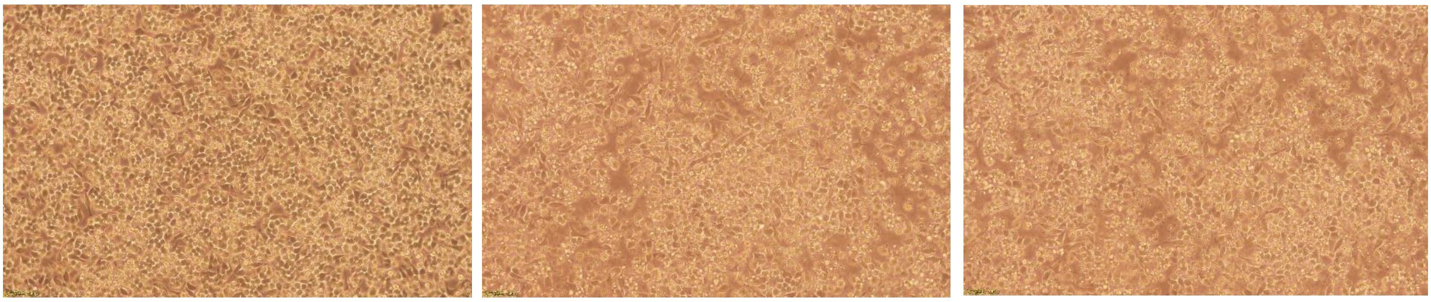

(A) (B) (C)

Figure S2. Cytopathic effect of RAW264.7 infected with CA09 and SD56. (A) RAW264.7 cells of the uninfected group showed no obvious lesions. (B) RAW264.7 cells in CA09 infected group showed obvious lesions. (C) RAW264.7 cells in SD56 infected group showed obvious lesions. Magnification is 200X.

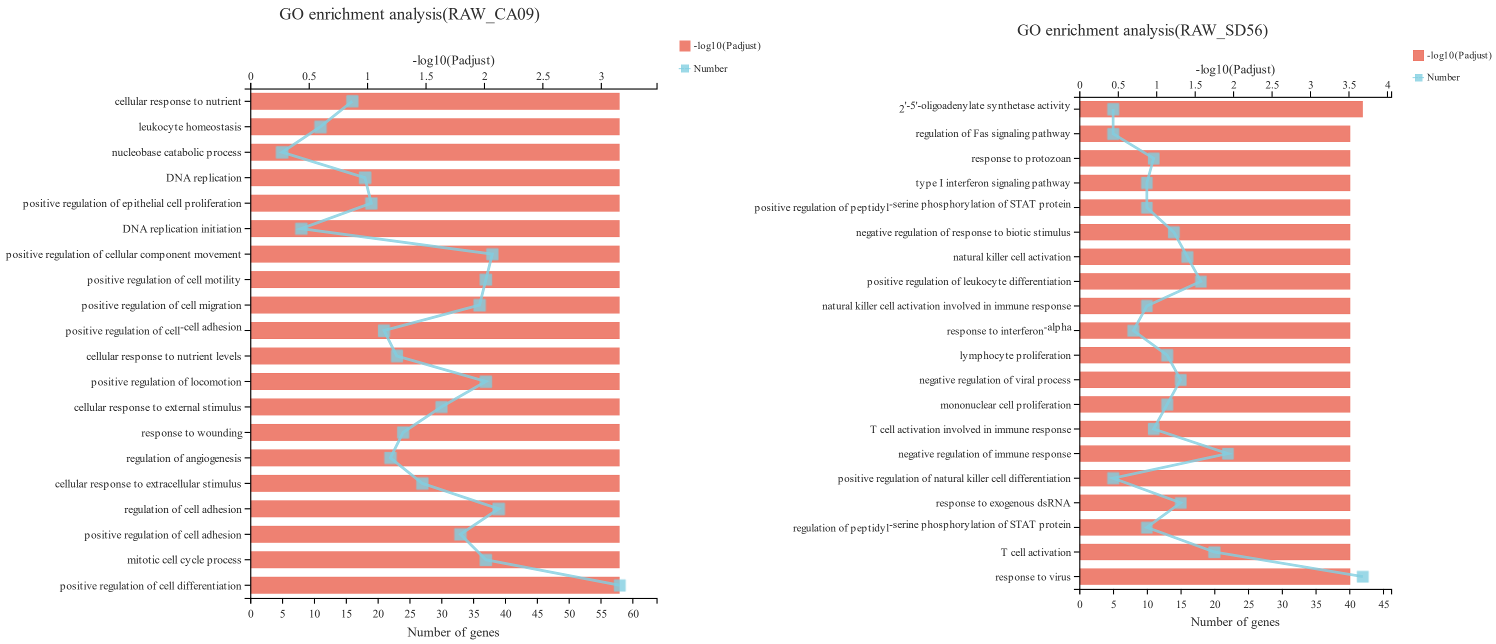

(A) (B)

Figure S3. GO functional enrichment of differentially expressed genes in RAW264.7.(A) GO analysis of RAW264.7 differentially expressed genes in CA09 relative to the control. (B) GO analysis of RAW264.7 differentially expressed genes in SD56 relative to the control.

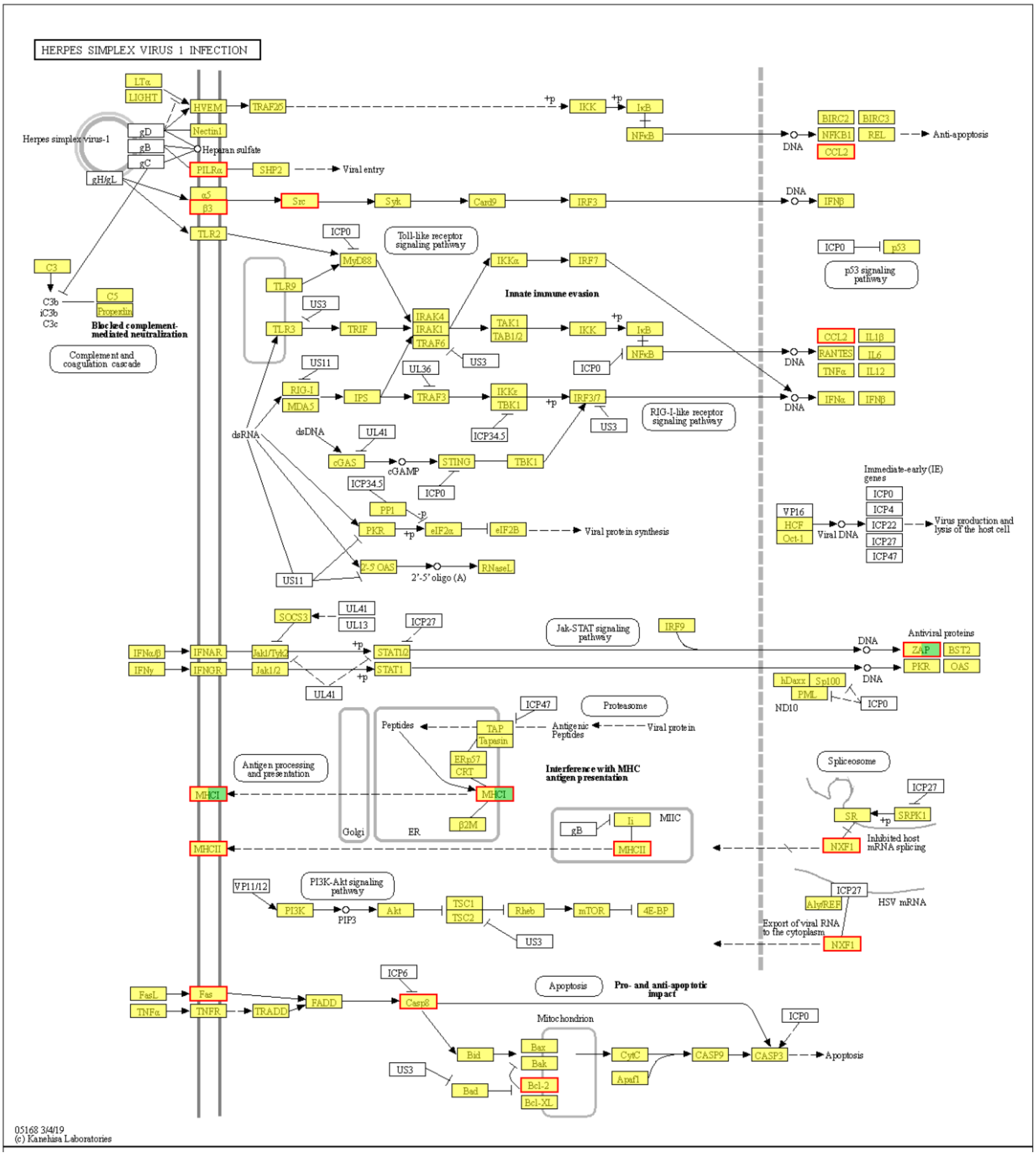
HERPES SIMPLEX VIRUS 1 INFECTION
LTα
LIGHT
HVEM
TRAF2/5
IKK
IκB
NFκB
Nectin1
Herpes simplex virus-1
gD
gB
gC
gH/gL
Heparan sulfate
PILRα
SHP2
Viral entry
α5
β3
Src
Syk
Card9
IRF3
IFNβ
TLR2
ICP0
MyD88
Toll-like receptor signaling pathway
IKKα
IRF7
C3
C3b
iC3b
C3c
C5
Properdin
Blocked complement-mediated neutralization
Complement and coagulation cascade
TLR9
US3
TLR3
TRIF
IRAK4
IRAK1
TRAF6
TAK1
TAB1/2
Innate immune evasion
US11
UL36
RIG-I
MDA5
IPS
TRAF3
IKKε
TBK1
IRF3/7
RIG-I-like receptor signaling pathway
ICP34.5
dsRNA
dsDNA
UL41
cGAS
cGAMP
STING
PP1
PKR
eIF2α
eIF2B
Viral protein synthesis
2'-5' OAS
2'-5' oligo (A)
RNaseL
BIRC2
BIRC3
NFKB1
REL
CCL2
Anti-apoptosis
DNA
p53
p53 signaling pathway
IL1β
RANTES
IL6
TNFα
IL12
IFNα
Immediate-early (IE) genes
ICP4
ICP22
ICP27
ICP47
VP16
HCF
Oct-1
Viral DNA
Virus production and lysis of the host cell
SOCS3
UL13
IFNα/β
IFNAR
Jak1/Tyk2
STAT1/2
Jak-STAT signaling pathway
IRF9
Antiviral proteins
ZAP
BST2
OAS
IFNγ
IFNGR
Jak1/2
STAT1
hDaxx
Sp100
PML
ND10
Proteasome
Peptides
TAP
Tapasin
Antigenic Peptides
Viral protein
ERp57
CRT
MHCI
Antigen processing and presentation
Interference with MHC antigen presentation
Spliceosome
SR
SRPK1
Golgi
ER
β2M
MIIC
Ii
MHCII
NXF1
Inhibited host mRNA splicing
VP11/12
PI3K-Akt signaling pathway
PI3K
PIP3
Akt
TSC1
TSC2
Rheb
mTOR
4E-BP
Aly/REF
HSV mRNA
Export of viral RNA to the cytoplasm
ICP6
FasL
Fas
FADD
Casp8
TNFR
TRADD
Apoptosis
Pro- and anti-apoptotic impact
Mitochondrion
Bid
Bax
Bak
Bcl-2
Bcl-XL
Bad
CytC
Apaf1
CASP9
CASP3
05168 3/4/19
(c) Kanehisa Laboratories

(A)

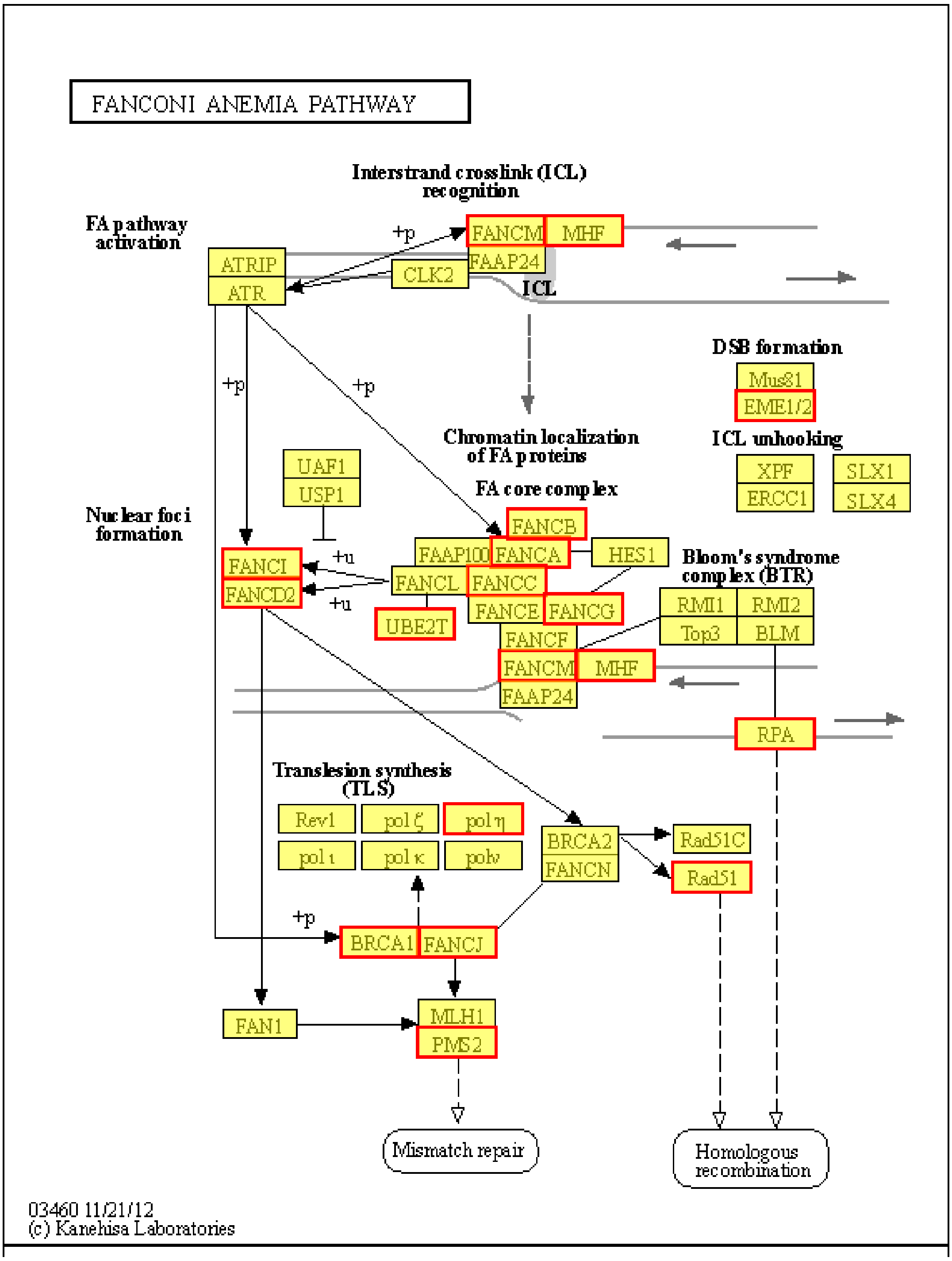

(B)

Figure S4. Details of the signaling pathways involved in modules.(A) Details of Herpes simplex virus 1 infection pathway and related up-regulation and down-regulation genes; (B) Details of Fanconi anemia pathway and related up-regulation and down-regulation genes.

Table S1 Transcriptome Profile of 18 cDNA libraries

SAMPLE	RawReads	Clean Reads	Clean Reads Percent	Q30%	Total mapped	Uniquely mapped	CDS
Lung_SD56_1	53146554	52566676	98.9%	91.4	50348605(95.8%)	47164508(89.7%)	52240657.0(70.7%)
Lung_ SD56_2	53212994	52615142	98.9%	91.2	50238054(95.5%)	47843180(90.9%)	52154841.0(70.3%)
Lung_ SD56_3	53307084	52756724	99.0%	91.4	50443422(95.6%)	47909895(90.8%)	53155786.0(71.0%)
Lung_CA09_1	53062352	52331376	98.6%	90.2	49578641(94.7%)	46892214(89.6%)	51168736.0(70.1%)
Lung_ CA09_2	53315812	52788194	99.0%	91.7	49662285(94.1%)	46977241(89.0%)	49972826.0(68.9%)
Lung_ CA09_3	53120896	52617316	99.1%	92.0	50302265(95.6%)	47608152(90.5%)	53701875.0(71.5%)
Lung_con_1	52604088	51936712	98.7%	94.0	49910648(96.1%)	46831341(90.2%)	48737823.0(68.0%)
Lung_con_2	47144746	46541456	98.7%	94.0	44688267(96.0%)	42000270(90.2%)	44211373.0(68.2%)
Lung_con_3	48534990	47880950	98.7%	93.9	45991411(96.1%)	43541960(90.9%)	44319805.0(67.1%)
RAW_ SD56_1	49532908	48881004	98.7%	92.8	43802836(89.6%)	41018374(83.9%)	48260720.0(73.2%)
RAW_ SD56_2	50516272	49835386	98.7%	92.9	44244362(88.8%)	41399847(83.1%)	48945530.0(73.4%)
RAW_ SD56_3	47781244	47106910	98.6%	92.6	41981169(89.1%)	39395090(83.6%)	46642537.0(73.5%)
RAW_ CA09_1	47438302	46738398	98.5%	93.0	43018095(92.0%)	40211896(86.0%)	49907335.0(75.4%)
RAW_ CA09_2	48729094	47862316	98.2%	92.5	44090155(92.1%)	41198705(86.1%)	51002728.0(75.3%)
RAW_CA09_3	46360036	45669944	98.5%	93.2	42239428(92.5%)	39487312(86.5%)	49595575.0(76.0 %)
RAW_con_1	45842734	45148086	98.5%	92.8	42524553(94.2%)	39921487(88.4%)	47702254.0(73.7%)
RAW_con_2	53672672	52976794	98.7%	93.2	49898508(94.2%)	46836772(88.4%)	55503054.0(73.3%)
RAW_con_3	49170826	48474162	98.6%	92.6	45447559(93.8%)	42660584(88.0%)	51177175.0(73.9%)

Raw reads: The original sequence data were counted in four units, and the number of sequencing sequences in each file was counted.

Clean reads: The number of sequenced data after filtering.

Clean reads percent: The ratio of clean reads in raw reads.

Q30: The percentage of bases with sequencing quality above 99.9% to total bases.

Total mapped: The number of clean reads that can be mapped to the genome.

Uniquely mapped: The number of clean reads with unique alignment positions on the reference sequence

CDS: The reads distributed in protein coding regions
